# A Pan-Cancer Transcriptomic Signature for Conserved Molecular Programs Underlying Premalignant–Malignant Progression Across Common Carcinomas

**DOI:** 10.3390/dj14040228

**Published:** 2026-04-13

**Authors:** Kimia Sadat Kazemi, Marta Miyazawa, João Adolfo Costa Hanemann, Marisa Ionta, Pollyanna Francielli de Oliveira, Andrew Leask, Cristiane Miranda Franca, Felipe Fornias Sperandio

**Affiliations:** 1 College of Dentistry, University of Saskatchewan, Saskatoon, SK S7N 5E5, Canada; kimia.kazemi@usask.ca (K.S.K.); anl312@mail.usask.ca (A.L.); 2 Faculdade de Odontologia, Universidade Federal de Alfenas, Alfenas 37130-001, MG, Brazil; marta.miyazawa@unifal-mg.edu.br (M.M.); jachanemann@unifal-mg.edu.br (J.A.C.H.); 3 Instituto de Ciências Biomédicas, Universidade Federal de Alfenas, Alfenas 37130-001, MG, Brazil; marisa.ionta@unifal-mg.edu.br; 4 Instituto de Ciências da Natureza (ICN), Universidade Federal de Alfenas, Alfenas 37130-001, MG, Brazil; pollyanna.oliveira@unifal-mg.edu.br; 5 Department of Biomaterial and Biomedical Sciences, School of Dentistry, Oregon Health & Science University, Portland, OR 97239-3098, USA; mirandaf@ohsu.edu

**Keywords:** oral squamous cell carcinoma (OSCC), oral potentially malignant disorders (OPMDs), pan-cancer transcriptomics, premalignant lesions, biomarker discovery, gene expression signatures, cancer-testis antigens (MAGEA family), transcriptional regulation, malignant transformation, early cancer detection

## Abstract

**Background/Objectives:** Oral squamous cell carcinoma (OSCC) commonly arises from oral potentially malignant disorders (OPMDs), yet reliable molecular biomarkers that predict malignant transformation remain scarce. Because epithelial carcinogenesis follows similar multistep trajectories across multiple organs, pan-cancer transcriptional analyses may reveal conserved pathways relevant to early oral tumorigenesis. This study aimed to identify shared transcriptional signatures across carcinomas and evaluate their applicability to precancerous-to-carcinoma progression. **Methods:** Bulk RNA-seq data from five carcinomas (lung, colon, breast, prostate, and head and neck squamous cell carcinoma, HNSCC) were obtained from TCGA to identify shared differentially expressed genes (DEGs) (|log_2_FC| ≥ 2; FDR < 0.05). Functional enrichment, clustering, and gene–pathway network analyses characterized conserved biological processes. Independent GEO datasets containing premalignant and malignant samples, including OPMD and OSCC cohorts, were examined to assess early-stage relevance. **Results:** A conserved 45-gene signature was identified, enriched for transcriptional regulation, chromatin organization, and RNA polymerase II-mediated processes. Regulatory hubs, including ZIC5, MYBL2, ONECUT2, POU4F1, and PDX1, and strong upregulation of cancer-testis antigens (MAGEA3, MAGEA6, MAGEC2) were notable. Integration with premalignant datasets revealed 13 genes consistently dysregulated across early lesions, involving pathways such as cell differentiation, apoptosis, and lipid transport. Several genes remained altered from normal tissue through OPMD to OSCC, supporting their potential as stable biomarkers. **Conclusions:** This study identifies conserved transcriptional programs shared across epithelial cancers and detectable in OPMDs. These findings highlight promising biomarker and regulatory candidates for improving early detection and risk stratification of oral precancer, addressing a critical unmet need in OSCC prevention and clinical management.

## 1. Introduction

Common human malignancies such as prostate, colorectal, lung, breast, and head and neck squamous cell carcinoma (HNSCC) constitute around 40% of worldwide cancer incidence and related mortality [1]. Although major advances have been achieved in surgical techniques, radiotherapy, and systemic treatment modalities, overall survival improvements for many of these tumor types remain limited, underscoring the need for a deeper understanding of the molecular processes that govern tumor initiation and disease progression [2]. Notably, many of these cancers develop through a gradual, multistep evolution originating from premalignant or precursor lesions. Canonical examples include the transition of sessile serrated lesions to colorectal adenocarcinoma and the progression of oral potentially malignant disorders (OPMDs) toward oral squamous cell carcinoma (OSCC), both of which illustrate the stepwise nature of epithelial carcinogenesis [3,4].

Population-based longitudinal studies and meta-analyses have demonstrated that the likelihood of malignant transformation correlates with the histopathological severity of dysplasia; however, reliance on histological grading alone remains problematic since significant inter-observer variability and limited prognostic resolution at the level of individual patients restrict its clinical utility [5]. In an optimal framework, the propensity for malignant progression would instead be inferred from defined molecular signatures, such as gene expression profiles capable of stratifying lesions according to transformation risk. Large-scale efforts like The Cancer Genome Atlas (TCGA) Pan-Cancer Analysis Project provide a valuable foundation for this approach by systematically cataloguing genomic and transcriptomic alterations across thousands of tumors spanning multiple tissue origins, thereby uncovering both shared oncogenic mechanisms and lineage-specific features [6].

Addressing this unmet need requires the identification of transcriptional signatures that transcend tissue specificity and capture conserved molecular programs associated with malignant progression. This challenge is particularly relevant in the context of OPMDs, where clinical examination and histopathological assessment often fail to reliably forecast cancer development. The discovery of common transcriptional drivers operative across diverse epithelial tissues could therefore facilitate earlier risk assessment, refine surveillance strategies, and support more individualized clinical management. Furthermore, delineating molecular programs that persist across both premalignant and malignant stages may yield insight into core biological processes underlying epithelial tumor initiation and evolutionary progression.

In this study, we systematically interrogated shared and cancer-specific gene expression programs across malignant tumors and their corresponding premalignant lesions. Our objective was to identify conserved transcriptional regulators, immune- and metabolism-associated nodes that function as recurrent drivers of epithelial carcinogenesis and inform strategies for early detection, risk stratification, and therapeutic targeting, with particular relevance on HNSCC. To this end, bulk RNA sequencing data from TCGA encompassing five major human carcinomas (lung, colon, breast, prostate, and HNSCC) were integrated with independent Gene Expression Omnibus (GEO) datasets representing premalignant and malignant disease stages. This integrative framework enabled the identification of (i) a core pan-cancer gene signature, (ii) transcriptional pathways consistently activated across tissues, and (iii) a subset of genes exhibiting sustained dysregulation from early lesions through invasive disease.

## 2. Materials and Methods

### 2.1. Analysis of Normal–Malignant Transition Using Public Cancer Genomic Datasets

Data Acquisition: RNA sequencing data were obtained from The Cancer Genome Atlas (TCGA) through the Genomic Data Commons (GDC) Data Portal. Five common human cancer types were included for pan-cancer analysis: lung adenocarcinoma (LUAD), prostate adenocarcinoma (PRAD), colon adenocarcinoma (COAD), breast invasive carcinoma (BRCA), and head and neck squamous cell carcinoma (HNSCC).

Raw HTSeq-count files were processed in R (version 2024.12.1+563) using edgeR (v3.44.0) and limma (v3.58.0). Genes with insufficient expression across samples were filtered using the filterByExpr function implemented in the edgeR package (v3.44.0). Library sizes were normalized using the trimmed mean of M-values (TMM) approach implemented in edgeR, followed by log2 counts-per-million transformation using the voom function to account for mean–variance relationships. edgeR/limma was used for normalization and visualization, whereas DESeq2 was applied for differential expression analysis to ensure robust statistical inference.

Differential Gene Expression Analysis: Differential expression was assessed separately for each cancer type using DESeq2 (v1.42.0). Raw counts were normalized using the median-of-ratios method, and tumor or premalignant samples were compared against matched normal tissues. Statistical significance was evaluated using Wald tests, with multiple testing correction performed using the Benjamini–Hochberg false discovery rate method. Genes with |log2FC| ≥ 2 and FDR < 0.05 were considered differentially expressed.

Construction of the Pan-Cancer Gene Signature: Differentially expressed genes from each cancer type were intersected using the VennDiagram package (v1.7.3). Genes consistently dysregulated in the same direction across all five cancers were retained as the core pan-cancer gene signature and subjected to downstream clustering and functional analyses.

Hierarchical Clustering and Inter-Cancer Similarity Visualization: Cosine similarity scores were calculated between normalized expression or enrichment vectors for each cancer type. Similarity matrices were visualized as clustered heatmaps using ComplexHeatmap (v2.19.0), with complete linkage hierarchical clustering and cosine distance (1 − cosine similarity). Analyses were performed using the coop, lsa (v0.74.1), and stats packages, with heatmaps scaled between 0.65 and 1.00.

Heatmap Visualization of Functional Annotation and Gene Expression Patterns: An integrated heatmap was generated to display scaled expression levels of the 45-gene pan-cancer signature alongside functional annotations. Expression values were visualized using a blue-to-red gradient, while gene involvement in selected GO terms or pathways was overlaid as a binary annotation. Visualizations were produced using ComplexHeatmap.

Functional Enrichment and Pathway Analysis: Gene Ontology enrichment analysis was conducted using clusterProfiler (v4.10.0) for biological processes, cellular components, and molecular functions. Overrepresentation was assessed using an FDR-adjusted *p*-value < 0.05. Cosine similarity matrices based on enrichment scores were generated and visualized using ComplexHeatmap and pheatmap.

Gene–Pathway Network Construction and Visualization: Gene–pathway interaction networks were constructed in Cytoscape (v3.10.2) based on GO enrichment results. Genes and GO terms were represented as nodes, with edges indicating functional associations. Gene nodes were color-coded by connectivity, enabling identification of highly connected hubs involved in transcriptional regulation and nuclear processes.

Correlation Analysis and Visualization: Inter-cancer similarities in biological process activity were quantified using cosine similarity applied to GO enrichment vectors. Heatmaps were generated with pheatmap (v1.0.12) and ComplexHeatmap, and clustering was performed using complete linkage. Cluster robustness was evaluated using pvclust and cophenetic correlation coefficients. Mantel tests (vegan package) were used to assess the statistical significance of similarity matrices following z-score normalization.

Software and Reproducibility: All analyses were conducted in R (version 2024.12.1+563). Analytical workflows were documented using RMarkdown and managed with Git version control. Figures were generated using ggplot2 (v3.5.0), pheatmap, and ComplexHeatmap, and finalized in Adobe Illustrator 2026 30.0.

### 2.2. Analysis of Premalignant-to-Malignant Transition Using GEO Datasets

Data Acquisition: Publicly available transcriptomic datasets were retrieved from the Gene Expression Omnibus (GEO) to investigate premalignant-to-malignant progression. Selected datasets included: GSE41657 (colon cancer), GSE227919 (HNSCC), GSE79210 and GSE132690 (lung cancer), and GSE34279 (breast cancer). Datasets were chosen based on sample size, availability of premalignant stages, and clear histological annotation. Prostate cancer was included in the TCGA-based pan-cancer analysis; however, it was not incorporated into the premalignant validation phase due to the absence of well-characterized premalignant datasets in GEO repositories.

Data Preprocessing and Normalization: Preprocessing methods were tailored to each platform. Microarray datasets were background-corrected and normalized using limma with RMA or quantile normalization, while RNA-seq datasets were normalized using DESeq2. Low-information genes or probes were filtered using count-based or interquartile range (IQR) thresholds.

Differential Gene Expression Analysis: Differential expression was assessed across disease stages within each dataset using limma or DESeq2, depending on data type. Comparisons included premalignant and malignant samples relative to normal tissues. Genes with |log2FC| ≥ 1.5 and adjusted *p*-value < 0.05 were defined as differentially expressed. A slightly relaxed threshold (1.5 rather than 2) was applied to GEO datasets to account for smaller sample sizes and increased biological heterogeneity.

Construction of Shared Progression-Associated Gene Signatures: Within each dataset, overlapping genes between premalignant-versus-normal and malignant-versus-normal comparisons were identified using the VennDiagram package. These gene sets were cross-referenced with the TCGA-derived 45-gene pan-cancer signature to identify early dysregulated genes, followed by functional annotation.

Identification and Clustering of Upregulated Core Genes: Genes consistently upregulated across all five TCGA cancer types were extracted and subjected to hierarchical clustering based on expression profiles across tumor and normal samples. Clustering was performed using complete linkage with an appropriate distance metric, and results were visualized using ComplexHeatmap.

Hierarchical Clustering and Similarity Visualization: Cosine similarity matrices were generated from GO enrichment profiles across GEO datasets. Hierarchical clustering using cosine distance and complete linkage was applied, and similarity patterns were visualized with ComplexHeatmap and pheatmap.

Heatmap Visualization of Functional Annotation and Expression Trends: Heatmaps were generated to visualize expression patterns of genes associated with selected GO biological processes across disease stages. Scaled expression values were displayed using a blue-to-red color gradient, with hierarchical clustering applied to both genes and samples.

Functional Enrichment Analysis: GO and KEGG pathway enrichment analyses were performed using clusterProfiler (FDR-adjusted *p* < 0.05), with a focus on pathways dysregulated during early tumorigenesis. Selected enrichment results were further examined using cross-cancer similarity analyses.

Gene–Pathway Network Construction: Gene–pathway networks were constructed in Cytoscape to visualize relationships between key differentially expressed genes and enriched biological processes. Nodes were color-coded by function and connectivity, and layouts were optimized using the Prefuse Force Directed algorithm.

Cross-Dataset Correlation and Comparative Analysis: Pairwise cosine similarity and Pearson correlation analyses were conducted using pathway enrichment profiles to compare datasets. Clustered heatmaps were generated to visualize shared biological programs, and Mantel tests were used to assess the statistical significance of similarity matrices.

Software and Reproducibility: All analyses were performed in R, with workflows documented using RMarkdown and managed through Git. Visualizations were generated using ggplot2, ComplexHeatmap, and seaborn. Network analyses were conducted in Cytoscape, and all figures were finalized in Adobe Illustrator.

## 3. Results

### 3.1. Gene Expression Signatures Among Common Human Cancers

**Figure 1:**
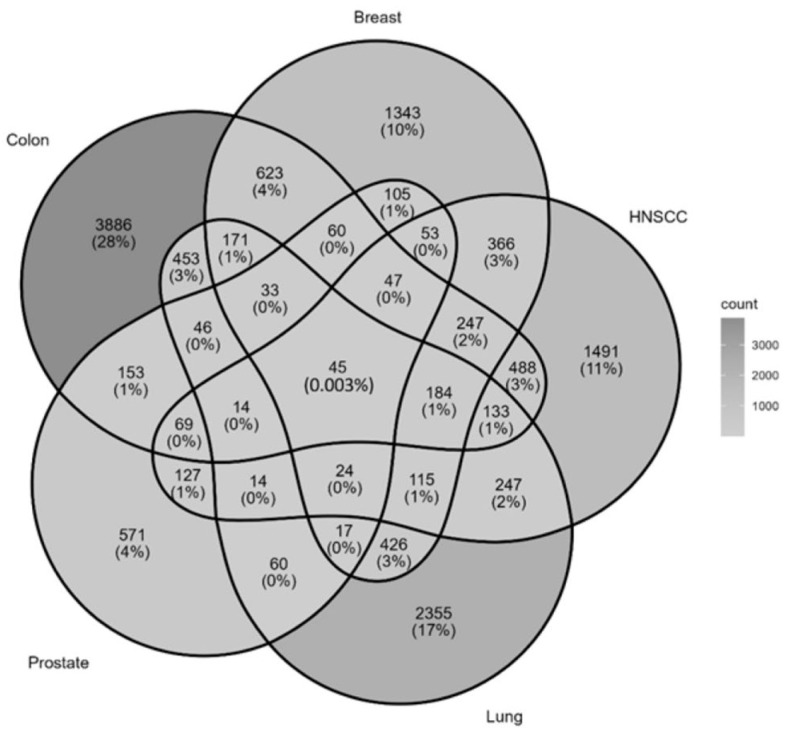
Overlap of gene expression signatures among colon, breast, HNSCC, prostate, and lung cancers. Each section represents the number and percentage of genes unique to or shared among different cancer types. Notably, 45 common genes are shared across all five cancers, highlighted in the central region (light grey). The color gradient indicates the count, with red representing higher counts and blue indicating lower counts, providing insights into common and distinct molecular pathways across these cancers.

The overlap of gene expression signatures among colon, breast, HNSCC, prostate, and lung cancers resulted in 45 common genes (stringent cutoff of |log_2_FC| ≥ 2) as depicted and listed in Figure 1.

**Figure 2:**
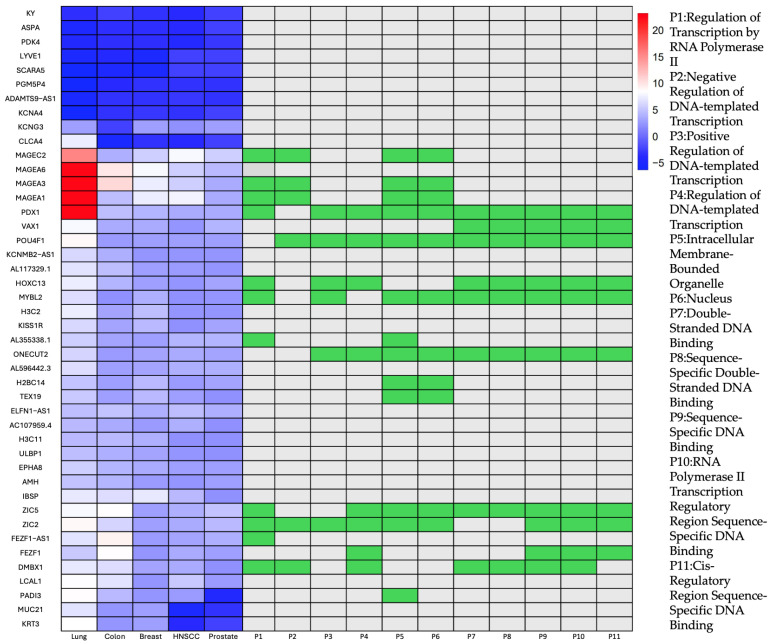
Expression levels of 45 candidate genes across five cancer types (lung, colon, breast, HNSC, and prostate) along with their functional associations to 11 enriched Gene Ontology (GO) biological processes (P1–P11). Blue to red gradients indicate relative gene expression (from low to high), and green squares represent pathway enrichment for each gene. Notable hub genes such as MYBL2, POU4F1, ZIC2, and ONECUT2 show strong correlations with transcriptional regulation pathways, while cancer/testis antigens (MAGEA1, MAGEA3) and VAX1 exhibit more restricted functional involvement. This integrative view highlights potential gene regulators implicated in transcriptional dysregulation across multiple cancer types.

The 45 interrelating genes and their corresponding relative expression in each of the studied cancer are described and illustrated on a heatmap in Figure 2. Here, the MAGEA gene family, encompassing MAGEA3, MAGEA6, and MAGEC2, demonstrated consistent and notable upregulation across all studied cancers with the highest expression observed in lung cancer (log_2_FC ≈ 22–23), followed by colon (log_2_FC ≈ 9–11), breast tumors (log_2_FC ≈ 7–8) and HNSCC (log_2_FC ≈ 5.66–7.98), indicating widespread activation of MAGE antigens across epithelial malignancies. The MAGE family encodes cancer-testis antigens normally restricted to germline tissues but reactivated in many tumors, where they regulate transcription, stress responses, and cell survival. Their tumor-restricted expression and immunogenicity make them important biomarkers and attractive targets for cancer immunotherapy [7].

**Figure 3:**
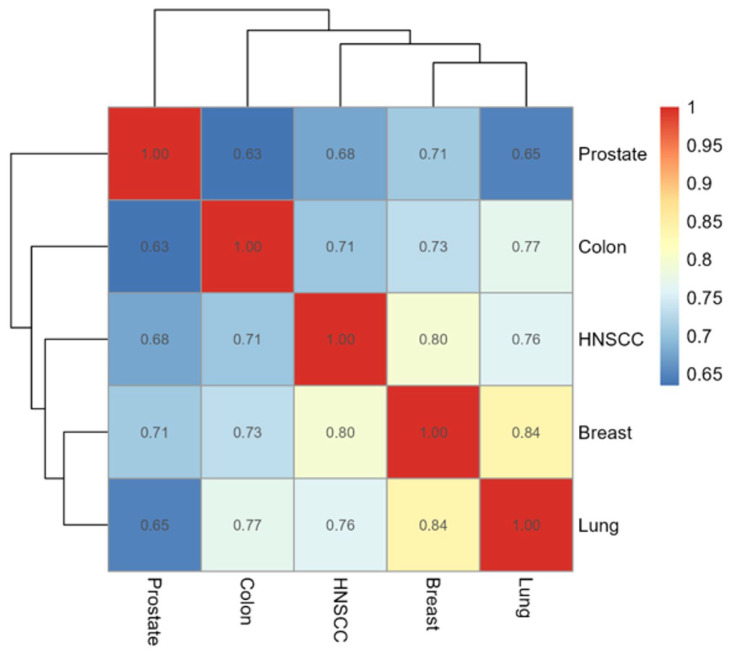
Pairwise correlation coefficients of gene expression profiles among HNSCC, prostate, lung, colon, and breast cancers. The color gradient ranges from blue (lower correlation) to red (higher correlation), with numerical values indicating the correlation coefficients. The hierarchical clustering on both axes reveals similarity patterns, showing that breast and lung cancers, for example, exhibit a higher correlation (0.84), whereas colon and prostate cancers have a lower correlation (0.63). This visualization helps identify cancer types with similar molecular signatures based on the expression of the 45 common genes.

A correlation heatmap helped quantify and visualize the degree of similarity in gene expression profiles between pairs of cancer types. The highest correlation coefficients obtained were between Breast and Lung (0.84), HNSCC and Breast (0.80), Colon and Lung (0.77), and HNSCC and Lung (0.76) (Figure 3).

### 3.2. Role of Overlapping Genes in Relevant Cell Signaling Pathways

Subsequent functional enrichment analysis using Gene Ontology (GO) databases was performed on the 45 genes identified. The enriched pathways are summarized in Table 1 to illustrate the key biological processes and signaling cascades implicated across multiple cancer types.

To assess transcriptional similarity in key biological pathways, pairwise cosine similarity scores were computed across five cancer types using enrichment scores derived from the shared 45-gene pan-cancer signature. As illustrated in the heatmaps (Figure A2), several Gene Ontology (GO) terms demonstrated consistently high inter-cancer similarity, reflecting conserved pathway activity across malignancies. Notably, “Positive Regulation of DNA-templated Transcription” (GO:0045893) and “Regulation of DNA-templated Transcription” (GO:0006355) exhibited strong cosine similarity scores (≥0.90) across all cancer types. Similarly, pathways associated with “Intracellular Membrane-Bounded Organelle” and “Nucleus” (GO:0043231, GO:0005634) revealed significant transcriptional resemblance. The strongest similarity was observed between HNSCC and breast cancer, particularly in pathways related to RNA Polymerase II activity and sequence-specific DNA binding. In contrast, lung cancer exhibited slightly lower similarity values in some pathways such as “Regulation of Transcription by RNA Polymerase II”.

### 3.3. Functional Stratification of Upregulated Core Genes

**Figure 4:**
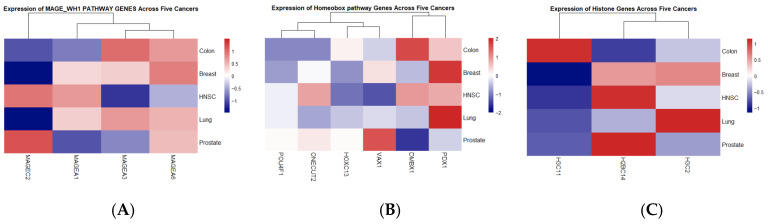
Pathway-specific heatmaps of upregulated pan-cancer genes. Heatmaps depict normalized expression (log_2_ fold change, tumor vs. normal) of consistently upregulated core genes belonging to (**A**) MAGE family domains (INTERPRO: MAGE_WH1), (**B**) homeobox transcription factor domains (UP_KW_DOMAIN: Homeobox), and (**C**) histone-associated chromatin components (INTERPRO: Histone H2A/H2B/H3) across five epithelial cancer types. Genes and cancer types were hierarchically clustered to highlight conserved and cancer-specific expression patterns.

Genes were further stratified based on directionality of dysregulation. Consistently upregulated genes were extracted for downstream functional enrichment analysis, as these genes are more likely to represent transcriptionally active oncogenic drivers. Enrichment analysis revealed three dominant functional modules among the upregulated core genes (Table 2), including MAGE family-associated domains (INTERPRO: MAGE_WH1), homeobox transcription factor domains (UP_KW_DOMAIN: Homeobox), and histone-related chromatin components (INTERPRO: Histone H2A/H2B/H3). To further illustrate the expression behavior of the enriched functional modules, pathway-specific heatmaps were generated for the three dominant categories identified in the enrichment analysis (Figure 4).

### 3.4. Gene Expression Signatures Among Premalignant Diseases

To evaluate whether the pathways identified from TCGA data were also relevant in the premalignant and progressive stages of each cancer, we analyzed transcriptomic datasets from the Gene Expression Omnibus (GEO) that include both premalignant and malignant samples. For colon cancer, dataset GSE41657 was analyzed. For HNSCC, dataset GSE227919 (OPMD, normal tissue, and OSCC) was used. For lung cancer, datasets GSE79210 (dysplasia vs. normal) and GSE132690 were included. For breast cancer, dataset GSE34279 (normal, premalignant, and malignant samples) was used. For prostate cancer, no direct premalignant dataset was available, and therefore prostate was excluded from further analysis.

**Figure 5:**
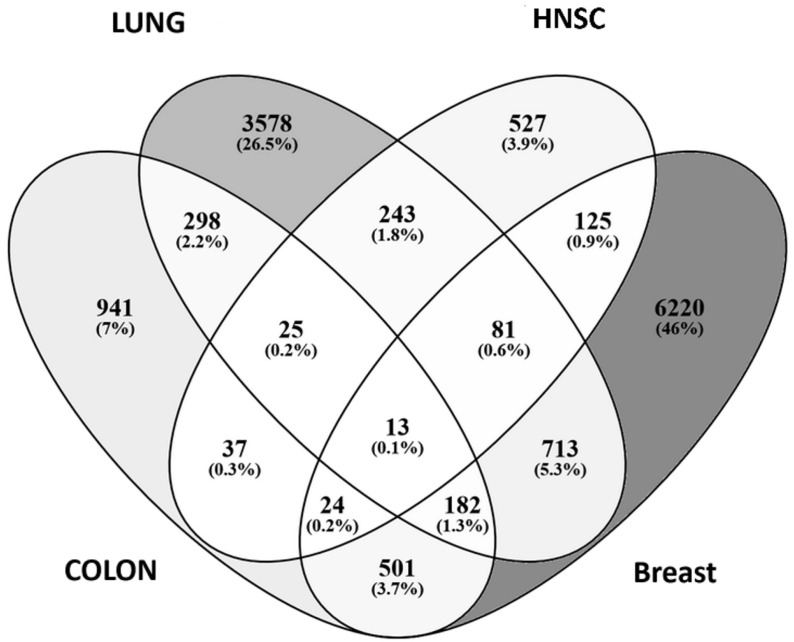
Overlap of differentially expressed genes (DEGs) among four cancer types: lung, colon, head and neck squamous cell carcinoma (HNSC), and breast cancer. Each segment displays the number and percentage of unique or shared DEGs. Breast cancer exhibits the highest number of unique DEGs (6220; 46%), followed by lung (3578; 26.5%). Shared gene sets across all four cancers include a small core (13 genes; 0.1%), suggesting a limited but potentially critical common molecular signature. The overlaps between two or three cancer types, such as lung and colon (298 genes; 2.2%) or colon and breast (182 genes; 1.3%), highlight possible shared oncogenic mechanisms across anatomical sites.

**Figure 6:**
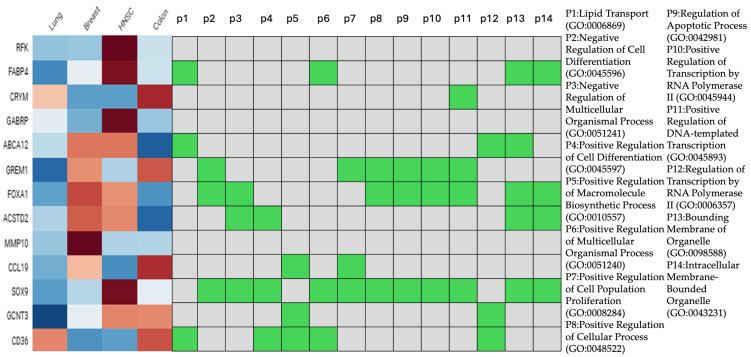
Expression levels of 13 candidate genes across four cancer types (lung, colon, breast, HNSCC) and their corresponding premalignant counterparts along with their functional associations to 14 enriched Gene Ontology (GO) biological processes (P1–P14). Blue to red gradients indicate relative gene expression (from low to high), and green squares represent pathway enrichment for each gene.

Overlaps and unique gene expression profiles among the lesions are illustrated in Figure 5; 13 genes (0.1%) were commonly dysregulated across all four cancer types: RFK, FABP4, CRYM, GABRP, ABCA12, GREM1, FOXA1, TACSTD2, MMP10, CCL19, SOX9, GCNT3, CD36. Breast cancer exhibited the highest number of unique DEGs (n = 6220, 46%), followed by lung cancer (n = 3578, 26.5%), then colon (n = 941, 7%) and HNSCC (n = 527, 3.9%). Additional overlaps were 243 genes between lung and breast, and 182 genes between colon and breast. To further characterize the shared transcriptional landscape across premalignant lesions, the 13 identified genes were plotted against a heatmap (Figure 6). Notable alterations were depicted in FABP4, GREM1, FOXA1, TACSTD2, SOX9, and CD36 (central nodes) across multiple cancer types. Functional enrichment analysis associated these genes with 14 Gene Ontology (GO) biological processes, including key regulatory mechanisms such as positive regulation of cell differentiation (GO:0045597), positive regulation of transcription by RNA polymerase II (GO:0045944), lipid transport (GO:0006869), and regulation of apoptotic process (GO:0042981) (Figure A3).

To evaluate the similarities in biological pathway activities across the different lesions, we performed a correlation analysis using enrichment scores derived from the 14 significantly overrepresented GO terms (Figure A4). Lipid transport pathway (GO: 0006869): the highest degree of similarity was observed between lung and colon cancers (similarity score: 0.75). In contrast, the overlap between breast cancer and HNSCC was moderate (0.5), while the lowest overlap was detected between breast and lung cancer (0.3). Negative Regulation of Cell Differentiation (GO:0045596): the highest degree of overlap was observed between lung and colon cancers (0.72). Moderate similarities are seen between HNSCC and lung (0.6) as well as HNSCC and colon (0.55). Breast cancer shows the lowest similarity with other cancers in this pathway, particularly with lung (0.3). Positive Regulation of Cell Differentiation (GO:0045597): there was a strong pathway similarity between HNSCC and both lung (0.86) and colon cancers (0.86). Breast cancer also shares a moderate similarity with lung and HNSCC (0.71). Colon and lung exhibit a lower correlation (0.57). Overall, HNSCC emerges as a central node in this network, showing the most consistent positive regulatory pathway similarity across other cancer types. Negative Regulation of Multicellular Organismal Process (GO:0051241): there were varying degrees of gene overlap among lesion types. The highest similarity was observed between breast cancer and HNSCC (similarity score: 0.85). HNSCC and colon cancer showed the lowest overlap (0.20). Lung and colon cancers shared moderate similarity (0.70). Positive Regulation of Macromolecule Biosynthetic Process (GO:0010557): the strongest similarity was observed between HNSCC and lung cancer (correlation coefficient: 0.92), followed by HNSCC and colon (0.86), and breast and lung (0.85). Breast and colon showed the lowest correlation (0.68). Overall, HNSCC demonstrated consistently high connectivity with other cancer types. Positive Regulation of Multicellular Organismal Process (GO:0051240): there was moderate to high correlations in gene expression profiles. Notably, HNSCC and colon cancer showed the strongest similarity (correlation coefficient: 0.85), followed by breast and lung cancers (0.80), suggesting potential conservation of developmental signaling mechanisms in these tissues. Lung and HNSCC, as well as lung and colon, exhibited moderate similarity scores (0.75 and 0.72, respectively), while breast and colon cancers demonstrated the lowest correlation (0.63), indicating some divergence in pathway activity.

**Figure 7:**
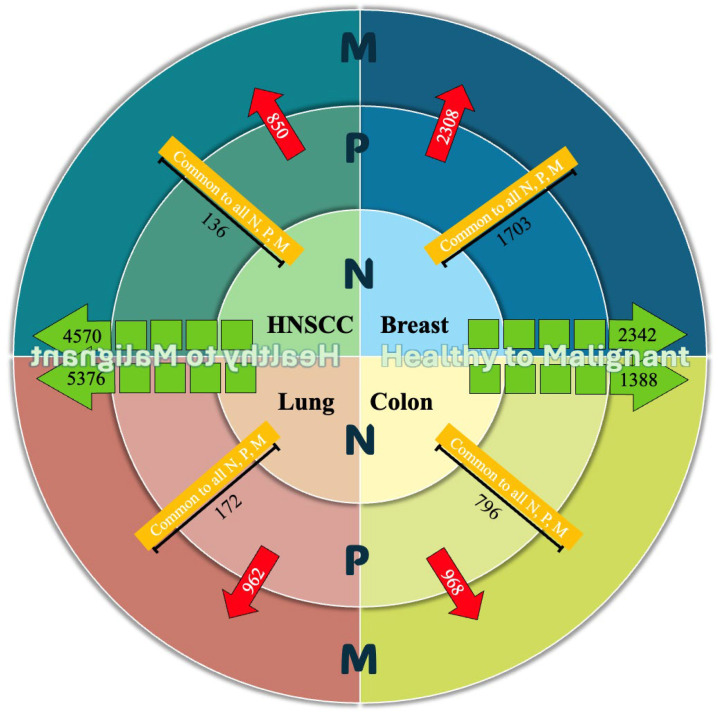
Comparison of gene expression dynamics across four carcinomas (HNSC, lung, colon, and breast) considering their preceding normal tissue and premalignant stages. Green arrows represent the number of genes directly dysregulated in the transition from healthy to malignant tissue. Yellow blocks indicate genes commonly expressed in all three stages: healthy, premalignant, and malignant. Red arrows show the number of genes specifically involved in the transition from premalignant to malignant stages. Comparison of gene expression dynamics across four epithelial cancers (HNSC, lung, colon, and breast).

Positive Regulation of Cell Population Proliferation (GO:0008284): there was substantial overlap in gene profiles among the four lesion types, with HNSCC and colon cancer exhibiting the highest similarity (correlation coefficient: 0.88), suggesting strong conservation of proliferative mechanisms. Breast and lung cancers also showed considerable similarity (0.83), while the lowest correlation was observed between breast and colon cancers (0.70). Positive Regulation of Cellular Process (GO:0048522): there were moderate to high correlation levels in gene expression across different lesions. HNSCC and lung cancer showed the strongest correlation (0.86), followed closely by HNSCC and colon (0.83), suggesting shared regulatory activity in increasing cellular functions such as proliferation, differentiation, migration and metabolism. Breast and lung cancer also displayed moderate similarity (0.78), while breast and colon cancers had the lowest correlation (0.67). These results imply that although the activation of cellular processes is a common hallmark across tumors, the degree of transcriptional overlap varies by tissue type, with HNSCC again showing high connectivity to lung and colon. Regulation of Apoptotic Process (GO:0042981): the highest similarity was noted between HNSCC and lung cancer (correlation coefficient: 0.81), indicating a shared apoptotic regulation profile. HNSCC and colon cancer also exhibited strong correlation (0.79), while the lowest was between breast and colon cancers (0.66). These findings suggest that while apoptotic control is a universal hallmark of cancer, the extent and mechanisms of regulation may differ by tumor origin, with epithelial cancers such as HNSCC and lung showing more conserved gene activity. Positive Regulation of Transcription by RNA Polymerase II (GO:0045944): there was a high similarity between HNSCC and lung cancer (correlation coefficient: 0.84), followed by HNSCC and colon cancer (0.82). Breast cancer showed lower correlations with colon (0.65) and HNSCC (0.70). Regulation of Transcription by RNA Polymerase II (GO:0006357): there were high correlations in gene expression profiles between HNSCC and lung cancer (0.85), and HNSCC and colon cancer (0.83). Breast cancer showed more modest correlations with HNSCC (0.73) and colon cancer (0.68). Bounding Membrane of Organelle (GO:0098588): there were notable correlations between HNSCC and lung lesions (0.82), with a slightly lower correlation to colon cancer (0.79). Breast cancer exhibited lower correlations with colon (0.66) and HNSCC (0.71). Positive Regulation of DNA-templated Transcription (GO:0045893): HNSCC and colon lesions demonstrated the strongest similarity (0.88), followed by lung (0.77). Breast lesions showed comparatively lower correlations with HNSCC (0.70) and colon cancer (0.65). To better understand the molecular overlap across lesion progression stages in different organs, a circular diagram was constructed (Figure 7). Cosine similarity reflects transcriptional concordance rather than direct mechanistic equivalence and should be interpreted with caution. High similarity may reflect either shared regulatory programs or broadly conserved oncogenic processes.

## 4. Discussion

Across the most common epithelial cancers, despite their diverse tissue origins, we identified a shared 45-gene transcriptional signature marked by MAGE cancer-testis antigen upregulation and a small subset of genes consistently dysregulated in premalignant lesions. These genes are associated with RNA polymerase II-driven transcription, cell differentiation, proliferation, and macromolecule biosynthesis with strong pathway similarity particularly between HNSCC and colon, supporting the emergence of conserved transcriptional programs early in epithelial carcinogenesis across tumors.

Within the 45-gene core signature, members of the MAGEA family (MAGEA3, MAGEA6, and MAGEC2) were consistently upregulated across carcinomas, with highest expression in lung and colon tumors and robust upregulation in HNSCC, where their expression has been linked to poor prognosis and immunogenicity [8,9]. Their recurrent activation across tumor types and presence in premalignant states support their role as tumor-associated antigens with potential diagnostic, prognostic, and immunotherapeutic relevance [10]. Consistent with this, MAGE proteins are established targets for vaccine and T-cell-based therapies due to their restricted expression in normal tissues and frequent activation in cancer [11,12,13]. In parallel, SOX9, another transcription factor identified in this analysis, has been implicated in shaping an immunologically “cold” tumor microenvironment and may influence responses to immunotherapy [14,15,16], further highlighting the translational relevance of these transcriptional regulators [17,18]. In fact, MAGE gene dysregulation has been associated with poorer survival in HNSCC [19], and mainly linked to negative regulation of transcription from RNA polymerase II, as well as negative regulation of protein processing [17].

A major strength of this study was the integration of TCGA with GEO datasets that captured premalignant stages of lung, colon, breast, and HNSCC. Only 13 genes were consistently dysregulated across premalignant lesions of all four cancers. Functional enrichment of these genes implicated key processes such as transcriptional regulation, lipid transport, and cell differentiation. Genes such as SOX9, GREM1, and TACSTD2 emerged as central regulators within the gene–pathway network, suggesting that dysregulation of cell fate and differentiation pathways is an early hallmark of malignant transformation across different tumors.

Correlation analysis of pathway activity across premalignant lesions revealed both conserved and divergent programs. For example, “positive regulation of cell differentiation” showed strong similarity between HNSCC, lung, and colon cancers (≥0.86), positioning HNSCC as a central node of convergence. In contrast, breast cancer consistently displayed weaker similarity to colon, lung, and HNSCC, indicating greater tissue-specificity in its transcriptional programs. Importantly, pathways related to apoptosis regulation and macromolecule biosynthesis also showed high cross-cancer similarity, underscoring their fundamental role in malignant transformation. These findings provided the foundation for downstream functional enrichment and network analyses to further characterize the shared biological processes and identify key hub genes involved in premalignant progression across cancers.

A major finding was that only a limited number of underscored genes persist across all disease stages. For instance, just 136 genes in HNSCC and 172 in lung cancer were consistently altered from premalignant through malignant stages. One can state that the molecular complexity of cancer progression often obscures early drivers, and only a restricted set of genes maintain relevance throughout disease evolution. Noticeably, these persistent dysregulated genes represent prime candidates for diagnostic and prognostic biomarker development.

Inter-cancer similarity was highlighted by strong correlations between breast and lung cancers (0.84) and moderate clustering of HNSCC with breast and lung. In contrast, prostate cancer exhibited the weakest correlations. While transcriptional dysregulation is a unifying hallmark, its magnitude and directionality are shaped by tissue-specific microenvironments and oncogenic exposures, such as smoking habits or other deleterious environmental behaviors [20,21,22]. For instance, genes involved in promoting cell population growth were commonly regulated across multiple tumor types, but with notable convergence between HNSCC and colon cancers: potential routes of similarity for carcinomas that originate in the GI tract (including oral cavity) thus appear to exist, and may guide future studies, i.e., investigation of the microbiome to understand the role of certain bacteria in the positive regulation of cell population and differentiation in HNSCC and colon cancers [23,24]. Moreover, the tissue specificity of individual cancer drivers may be affected by cell-extrinsic factors such as cell–cell signaling, mosaicism, and cooperation and competition between distinct cell types in the context of the respective tumor micro- and macroenvironments, as well as other environmental factors [25].

The network visualization revealed a dense connectivity pattern among genes involved in Regulation of Transcription by RNA Polymerase II, Positive/Negative Regulation of DNA-templated Transcription, Sequence-Specific DNA Binding, Intracellular Membrane-Bounded Organelle. Notably, genes ZIC5 and MYBL2 displayed the highest levels of connectivity, indicating their potential roles as central regulators in the molecular framework. Several genes like PDX1, POU4F1, ONECUT2 were linked to multiple transcription-related pathways, such as “Regulation of Transcription by RNA Polymerase II” and “Positive Regulation of DNA-Templated Transcription”, suggesting their pleiotropic regulatory functions. This network highlights the convergence of multiple cancer-associated genes on shared biological processes, particularly those related to transcriptional control and chromatin architecture, which further validates the relevance of a pan-cancer gene signature in capturing targeting alternatives for diagnosis and treatment.

Functional enrichment analysis reinforced the centrality of transcriptional regulation. Within the gene–pathway interaction network, ZIC5 and MYBL2 appeared as highly connected hubs, while POU4F1, ONECUT2, and PDX1 linked multiple transcriptional pathways. Interestingly, a relatively small number of transcriptional regulators were shown to orchestrate broad oncogenic programs across cancers, which make them attractive candidates for therapeutic targeting. Consonantly, ZIC5 has been implicated in promoting aggressiveness and stemness in melanoma and cervical SCC [26,27], while MYBL2 is increasingly recognized as an oncogenic regulator in lung adenocarcinoma and prostate cancer [28,29,30]. ONECUT2 has been established as a therapeutic target in castration-resistant prostate cancer, with the small-molecule inhibitor CSRM617 and related compounds showing robust suppression of tumor growth and metastasis in preclinical models [31,32]. Similarly, POU4F1 has emerged as a prognostic and therapeutic node across multiple malignancies, including basal-like breast cancer, esophageal SCC and melanoma, where its inhibition can restore sensitivity to endocrine or BRAF-targeted therapies [10,33,34].

Interestingly, long non-coding RNAs (lncRNAs) such as ELFN1-AS1 and LCAL1 showed robust upregulation in HNSCC. LCAL1 had been previously implicated in lung adenocarcinoma immunotherapy response [35], but was identified here for the first time in HNSCC. These observations suggest a potential relevance of lncRNAs as biomarkers and therapeutic targets in head and neck malignancies. In lung cancer, ELFN1-AS1 has been shown to promote proliferation, cell-cycle progression and EMT through ceRNA activity, while LCAL1 has been incorporated into immune- and metabolism-related prognostic models capable of predicting response to immune checkpoint inhibitors [35,36,37,38]. Their consistent overexpression in our cohort highlights the possibility that targeting these lncRNAs through antisense oligonucleotides, siRNA-mediated silencing, or CRISPR-interference approaches could modulate oncogenic networks and potentially enhance responsiveness to immunotherapy [39,40]. Future functional studies in HNSCC models will be essential to determine whether these lncRNAs act as drivers of immune dysfunction or therapeutic sensitizers, but their emergence across multiple epithelial cancers underscores their value as promising RNA-based nodes for therapeutic exploration.

MUC21 and KRT3 showed a distinct expression pattern that contrasted with the broadly conserved pan-cancer transcriptional program. Both genes were markedly downregulated in HNSCC but upregulated in lung, colon, and breast cancers, highlighting tissue-specific divergence within epithelial carcinogenesis. While recognized by its important protection role against pathogens in the oral mucosa [18,41], its differential expression has been noted in laryngeal squamous cell carcinoma as a downregulated gene (down-DEG) [42]. However, although recent studies have investigated the role of MUC21 in laryngeal SCC [42,43], it has not been extensively inspected in oral squamous cell carcinoma (OSCC). Given their roles in mucosal barrier function and epithelial differentiation, this opposing pattern suggests that, alongside shared oncogenic activation, loss of tissue-specific epithelial identity may be a defining feature of head and neck tumorigenesis and a potential avenue for early detection in oral and premalignant disease. Similarly, KRT3, a keratin gene typically associated with epithelial differentiation, exhibited substantial downregulation in HNSCC (log_2_FC = −3.75), while showing marked upregulation in lung cancer (log_2_FC = 8.45) and moderate increases in breast (2.80) and colon (2.29) cancers. Notably, KRT3 has been identified as a potential biomarker and therapeutic target for oral premalignant lesions [44]. These contrasting patterns may reflect lineage-specific epithelial differentiation programs; however, potential dataset-specific biases cannot be completely overlooked, and functional validation is required to confirm their roles in cancer progression.

The identification of a pan-cancer signature that is reproducible across both malignant and premalignant states offers a robust foundation for biomarker development and therapeutic intervention. Nevertheless, the heterogeneous degree of conservation observed among cancer types underscores the need to integrate pan-cancer frameworks with tissue-specific analyses. Moreover, the contribution of additional driver events not captured by the current analytical approach cannot be excluded [45]. These findings suggest that early detection and therapeutic strategies will likely need to be tailored to the distinct evolutionary trajectories of individual cancers.

Study limitations: Accordingly, this study is limited by its reliance on bulk transcriptomic data, which may obscure cell-type-specific contributions within heterogeneous tumor microenvironments. The identified 45-gene signature shows promise, but its clinical utility for risk stratification remains to be validated in independent cohorts and prospective studies to confirm the roles of candidate genes in tumor initiation and progression. Differences in threshold selection for the DEG analyses may have influenced comparability between analytical phases. Also, the absence of premalignant prostate datasets represents a limitation, as the relevance of prostate-derived genes to early tumorigenesis could not be evaluated. Future work should expand this analysis to include proteomic, epigenomic, and spatial transcriptomics approaches to better resolve the complexity of carcinogenesis, and prospective validation in cohorts of patients with oral dysplasia or OPMD will be essential to establish clinical utility.

## 5. Conclusions

In summary, this integrative analysis defines a conserved transcriptional signature shared across major epithelial cancers and shows that only a small fraction of genes remains consistently dysregulated from normal tissue through premalignant lesions to invasive disease. Although thousands of DEGs were detected between normal and malignant tissues (5376 in lung and 4570 in HNSCC), only 172 genes in lung cancer and 136 in HNSCC remained persistently dysregulated across all stages, with modest overlap between premalignant and malignant lesions (962 in lung and 850 in HNSCC). This sharp reduction highlights a critical bottleneck in identifying robust early biomarkers and underscores the need to prioritize these stage-spanning genes for validation in clinically relevant tissues, particularly OPMD and oral dysplasia, to support prevention, early detection, and timely intervention.

## Figures and Tables

**Table 1:** GO Pathway Enrichment Analysis of 45 Common Genes Across Cancer Types.

Category	Pathway/GO Term	Genes	Enrichment Score
Biological Process	Regulation of Transcription by RNA Polymerase II	DMBX1; ONECUT2;ZIC2;MAGEA1;PDX1;MYBL2;HOXC13;MAGEC2;ZIC5;FEZF1;POU4F1;MAGEA3	3.37
	Negative Regulation of DNA-templated Transcription	DMBX1;ZIC2;MAGEA1;MAGEC2;POU4F1;MAGEA3	2.93
	Positive Regulation of DNA-templated Transcription	ONECUT2;ZIC2;PDX1;MYBL2;HOXC13;POU4F1	2.38
	Regulation of DNA-templated Transcription (GO:0006355)	DMBX1;ONECUT2;ZIC2;PDX1;HOXC13;ZIC5;FEZF1;POU4F1	2.09
Cellular Component	Intracellular Membrane-Bounded Organelle (GO:0043231)	ONECUT2;PDX1;ZIC5;POU4F1;TEX19;H2BC14;ZIC2;KISS1R;MAGEA1;PADI3;MYBL2;MAGEC2;MAGEA3	1.20
	Nucleus (GO:0005634)	H2BC14;ONECUT2;ZIC2;MAGEA1;PDX1;MYBL2;MAGEC2;ZIC5;POU4F1;MAGEA3;TEX19	1.15
Molecular Function	Double-Stranded DNA Binding (GO:0003690)	DMBX1;ONECUT2;PDX1;MYBL2;HOXC13;ZIC5;VAX1;POU4F1	6.68
	Sequence-Specific Double-Stranded DNA Binding (GO:1990837)	DMBX1;ONECUT2;PDX1;MYBL2;HOXC13;ZIC5;VAX1;POU4F1	6.05
	Sequence-Specific DNA Binding (GO:0043565)	DMBX1;ONECUT2;PDX1;MYBL2;HOXC13;ZIC5;VAX1;POU4F1	6.03
	RNA Polymerase II Transcription Regulatory Region Sequence-Specific DNA Binding	DMBX1;ONECUT2;ZIC2;PDX1;MYBL2;HOXC13;ZIC5;VAX1;FEZF1;POU4F1	4.53
	Cis-Regulatory Region Sequence-Specific DNA Binding	ONECUT2;ZIC2;PDX1;MYBL2;HOXC13;ZIC5;VAX1;FEZF1;POU4F1	4.45
	RNA Polymerase II Cis-Regulatory Region Sequence-Specific DNA Binding	ONECUT2;ZIC2;PDX1;MYBL2;HOXC13;ZIC5;VAX1;FEZF1;POU4F1	4.35

**Figure A1:**
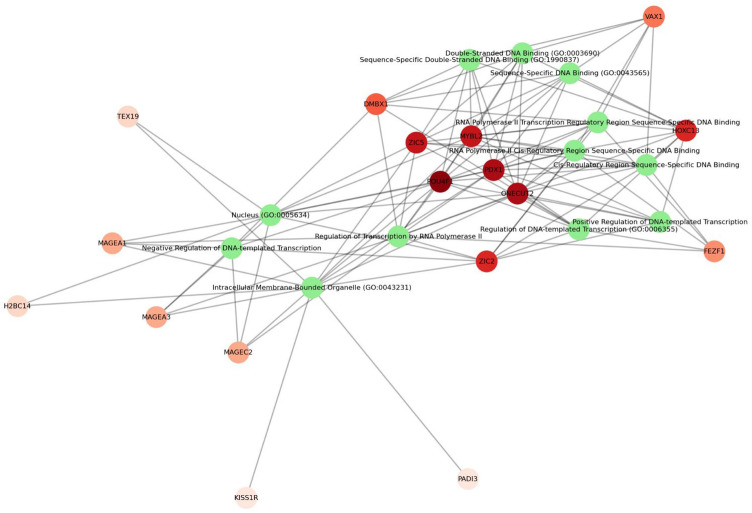
Gene–pathway interaction network displaying the functional associations between 17 candidate genes (nodes in shades of red) and enriched GO biological processes (green nodes). The intensity of red indicates gene connectivity, with hub genes such as ZIC5, MYBL2, POU4F1, and ONECUT2 showing multiple pathway associations. Edges represent functional involvement of each gene in the respective pathway, highlighting convergent roles in transcriptional regulation and DNA-binding processes.

**Figure A2:**
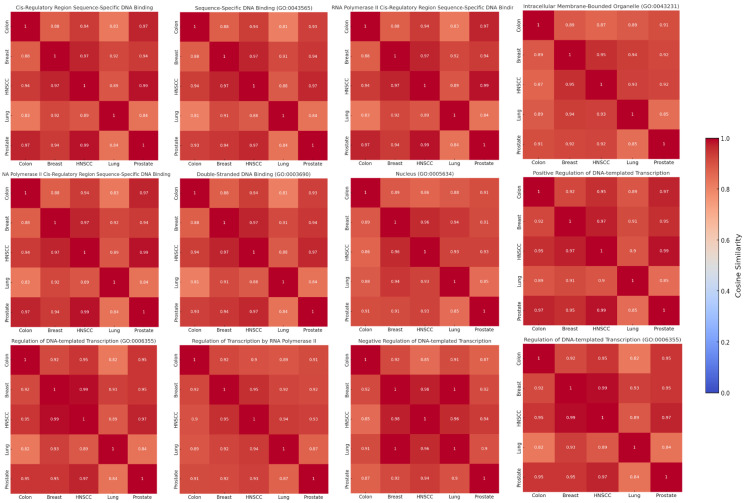
Cosine similarity heatmaps of enriched biological pathways across five epithelial cancers. Pairwise cosine similarity scores were calculated between colon, breast, HNSCC, lung, and prostate cancers for selected Gene Ontology (GO) biological processes enriched in the shared 45-gene pan-cancer signature. Each heatmap represents one pathway, with higher values (closer to 1.0, in red) indicating greater transcriptional similarity in pathway activity between cancer types. Prominent similarity was observed in transcription-related processes (e.g., “Regulation of DNA-templated Transcription”) and nuclear-associated components, suggesting conserved regulatory programs across distinct cancer tissues.

**Figure A3:**
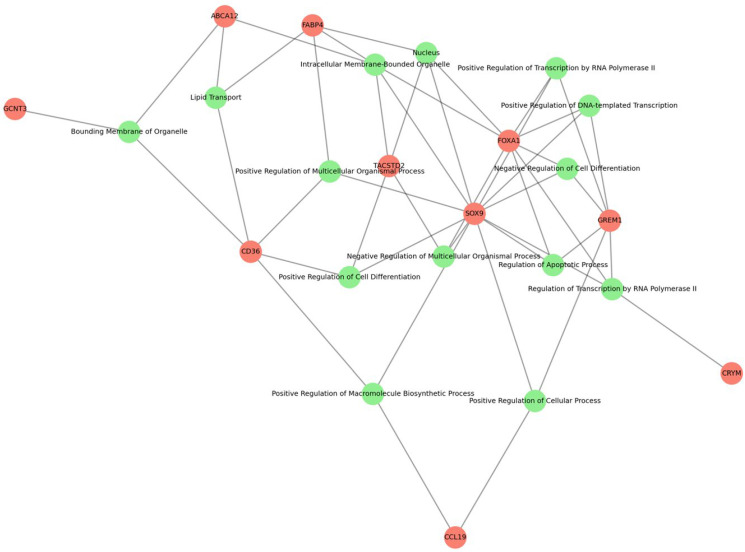
Gene–pathway network generated from enrichment analysis results. Red nodes represent genes, and green nodes denote significantly enriched biological pathways. The network highlights key regulatory relationships, with SOX9, FOXA1, and TACSTD2 emerging as central hub genes interacting with multiple pathways such as “Positive Regulation of DNA-Templated Transcription,” “Multicellular Organismal Process,” and “Apoptotic Process.” This network visualization emphasizes the multifaceted roles of select genes in driving transcriptional and developmental processes.

**Figure A4:**
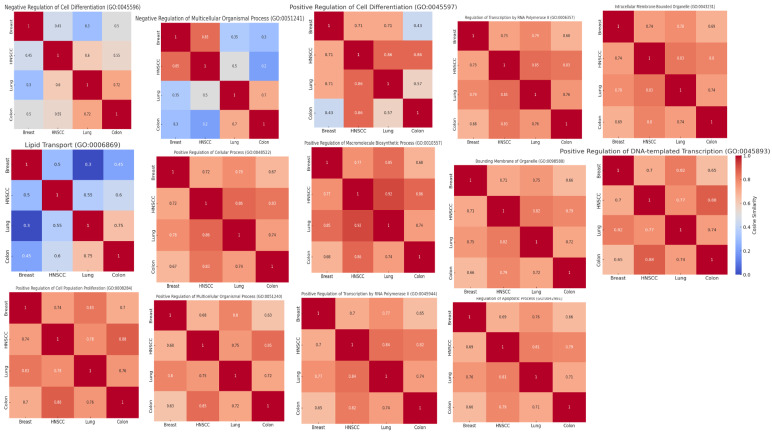
Cosine similarity heatmaps of selected Gene Ontology (GO) terms across four cancer types (Breast, HNSCC, Lung, and Colon). Each heatmap represents the similarity of gene sets enriched for specific GO biological processes or cellular components across different cancers. Notable patterns of high similarity (e.g., HNSCC–Lung in multiple GO terms) and low similarity (e.g., Breast–Colon in lipid transport) reveal shared and distinct functional landscapes among cancer types. GO terms include biological processes such as transcriptional regulation, cell differentiation, proliferation, apoptosis, and lipid transport, as well as subcellular components such as organelle membranes. The analysis highlights convergent and divergent molecular programs underpinning tumor biology.

To better understand the functional context of the identified gene set, a Gene–Pathway Correlation Network was constructed, consisting of 17 genes (only the genes that were associated with a given GO pathway, as extracted from the heatmap in Figure 2, and 11 enriched biological pathways (Figure A1).

**Table 2:** Functional enrichment of consistently upregulated core pan-cancer genes.

Pathway	Genes	Enrichment Scores
INTERPRO, MAGE_WH1	MAGEA1, MAGEA3, MAGEA6, MAGEC2	3.76
UP_KW_DOMAIN, Homeobox	POU4F1, DMBX1, HOXC13, ONECUT2, PDX1, VAX1	3.16
INTERPRO, Histone_H2A/H2B/H3	H2BC14, H3C11, H3C2	1.72

Summarized top enriched functional domains, including MAGE family-associated domains (INTERPRO: MAGE_WH1), homeobox transcription factor domains (UP_KW_DOMAIN: Homeobox), and histone-related chromatin components (INTERPRO: Histone H2A/H2B/H3). Listed genes represent members contributing to each enriched category, and enrichment scores reflect the relative strength of overrepresentation within the upregulated gene subset.

## Data Availability

The original contributions presented in this study are included in the article. Further inquiries can be directed to the corresponding author.
